# FIB/SEM technology and high-throughput 3D reconstruction of dendritic spines and synapses in GFP-labeled adult-generated neurons

**DOI:** 10.3389/fnana.2015.00060

**Published:** 2015-05-21

**Authors:** Carles Bosch, Albert Martínez, Nuria Masachs, Cátia M. Teixeira, Isabel Fernaud, Fausto Ulloa, Esther Pérez-Martínez, Carlos Lois, Joan X. Comella, Javier DeFelipe, Angel Merchán-Pérez, Eduardo Soriano

**Affiliations:** ^1^Developmental Neurobiology and Regeneration Unit, Department of Cell Biology and Parc Cientific de Barcelona, University of BarcelonaBarcelona, Spain; ^2^Centro de Investigación Biomédica en Red sobre Enfermedades Neurodegenerativas (CIBERNED), Insituto de Salul Carlos IIIMadrid, Spain; ^3^Institut de Recerca de l'Hospital Universitari de la Vall d'Hebron (VHIR)Barcelona, Spain; ^4^Laboratorio Cajal de Circuitos Corticales, Centro de Tecnología Biomédica, Universidad Politécnica de Madrid, Campus de MontegancedoMadrid, Spain; ^5^Instituto Cajal (Consejo Superior de Investigaciones Científicas)Madrid, Spain; ^6^Department of Neurobiology, University of Massachusetts Medical SchoolWorcester, MA, USA; ^7^Institut de Neurociències, Departament de Bioquímica i Biologia Molecular, Facultat de Medicina, Universitat Autònoma de BarcelonaBellaterra, Spain; ^8^Departamento de Arquitectura y Tecnología de Sistemas Informáticos, Escuela Técnica Superior de Ingenieros Informáticos, Universidad Politécnica de MadridMadrid, Spain; ^9^Institució Catalana de Recerca i Estudis Avançats AcademiaBarcelona, Spain

**Keywords:** dendritic spines, synapses, 3D-reconstruction, electron microscopy, FIB/SEM, adult neurogenesis

## Abstract

The fine analysis of synaptic contacts is usually performed using transmission electron microscopy (TEM) and its combination with neuronal labeling techniques. However, the complex 3D architecture of neuronal samples calls for their reconstruction from serial sections. Here we show that focused ion beam/scanning electron microscopy (FIB/SEM) allows efficient, complete, and automatic 3D reconstruction of identified dendrites, including their spines and synapses, from GFP/DAB-labeled neurons, with a resolution comparable to that of TEM. We applied this technology to analyze the synaptogenesis of labeled adult-generated granule cells (GCs) in mice. 3D reconstruction of dendritic spines in GCs aged 3–4 and 8–9 weeks revealed two different stages of dendritic spine development and unexpected features of synapse formation, including vacant and branched dendritic spines and presynaptic terminals establishing synapses with up to 10 dendritic spines. Given the reliability, efficiency, and high resolution of FIB/SEM technology and the wide use of DAB in conventional EM, we consider FIB/SEM fundamental for the detailed characterization of identified synaptic contacts in neurons in a high-throughput manner.

## Introduction

Adult neurogenesis has been described in most mammalian species (Lois and Alvarez-Buylla, 1994; Eriksson et al., 1998; Gage, 2000; Deng et al., 2010; Knoth et al., 2010; Sanai et al., 2011; Spalding et al., 2013), in two brain regions: the subventricular zone (SVZ) of the lateral ventricles and the subgranular zone (SGZ) of the dentate gyrus (DG). The new neurons continuously generated in these areas later differentiate and become integrated into functional circuits of the olfactory bulb and hippocampus, respectively. Hippocampal adult neurogenesis in mice exhibits a highly accurate temporal development, which has been precisely studied with the help of retrovirally-labeled synchronous neurogenic populations (Zhao et al., 2006; Ge et al., 2007) among other techniques. In brief, new neurons born in the SGZ migrate to the inner granule cell layer during their first week of age. At 2 weeks they already have a neuron-like morphology and receive depolarizing GABAergic input from interneurons in the granular layer (Ge et al., 2006). It has been described that 3-week-old granule cells (GCs) then start becoming integrated into their local network: their dendrites reach the molecular layer, where they receive glutamatergic excitatory input from entorhinal cortex axons. At the same time, hyperpolarizing events are triggered by GABAergic input to their somata (Ge et al., 2006, 2008). At this stage, these cells also exhibit mossy fiber boutons that establish efferent synaptic contacts with CA3 pyramidal cells (Sun et al., 2013). From 4 to 6 weeks, newborn GCs undergo a critical period during which they show stronger plasticity than mature GCs, both in terms of increased amplitude of LTP and a lower threshold for LTP induction (Ge et al., 2007). Finally, 8-week-old newborn granule cells exhibit synaptic plasticity parameters identical to those of mature granule cells, even though some features related to structural plasticity take longer to display mature phenotypes (Toni et al., 2007; Toni and Sultan, 2011). Strikingly, the morphology of dendritic spines (for simplicity, spines) in these neurons has been shown to change in response to environmental enrichment (Zhao et al., 2014), thereby suggesting a direct relationship between structure and function of newborn GC spines.

The fine dissection of microcircuits is essential for understanding normal brain function and for identifying structural and physiological modifications associated with neural plasticity and neuropathological conditions. The development of transmission electron microscopy (TEM) allowed the first fine analysis of synapses and revealed the high structural synaptic complexity of the nervous system (Peters et al., 1991; Peters and Palay, 1996). A further breakthrough was the combination of TEM with single neuron tracing methods (Golgi method, intracellular filling, etc.), which allowed the study of synaptic connectivity of identified neurons (Fairen et al., 1977; Somogyi and Hodgson, 1985; Frotscher and Leranth, 1986; Fairen, 2005). Although these techniques have provided fundamental information, the requirement of performing observations in ~60-nm ultrathin sections limits data analysis to a fragmented visualization as a result of the complex neuronal architecture. Efforts to successfully overcome this problem include analyzing serial ultrathin sections, which offers the possibility to reconstruct dendritic and axonal segments (Stevens et al., 1980; Harris et al., 2006; Arellano et al., 2007; Hoffpauir et al., 2007; Jain et al., 2010; Mishchenko et al., 2010; Bock et al., 2011). Obtaining series of such sections is extremely time-consuming and technically demanding, often making it impossible to reconstruct large volumes of tissue. Hence, the recent development of automated EM techniques is another crucial step for the study of synaptic contacts (Denk and Horstmann, 2004; Briggman and Denk, 2006; Knott et al., 2008; Merchan-Perez et al., 2009; Helmstaedter, 2013).

The combined use of focused ion beam milling (FIB) and scanning electron microscopy (SEM) has proven to be very useful for the study of brain ultrastructure (Knott et al., 2008; Merchan-Perez et al., 2009; Bushby et al., 2011; Peddie and Collinson, 2014). Furthermore, there is an increasing interest in using this technique to address correlative light and electron microscopy studies (Sonomura et al., 2013; Cane et al., 2014; Maco et al., 2014). Using FIB/SEM, synapses can be accurately identified, reconstructed and quantified (Merchan-Perez et al., 2009; Morales et al., 2011; Allegra Mascaro et al., 2013; Blazquez-Llorca et al., 2013; Maco et al., 2013; Sonomura et al., 2013). Here, we show that FIB/SEM technology reliably allows high-throughput 3D reconstruction of identified dendritic segments, spines, and input synapses from GFP-traced neurons, providing a resolution comparable to that of conventional TEM. We applied a correlative light microscope-FIB/SEM method to study developing synaptic inputs in retrovirally traced adult-generated granule cells (GCs). Adult neurogenesis and the recruitment of these neurons into the preexisting circuits are essential for learning and memory (Zhao et al., 2008; Deng et al., 2010; Southwell et al., 2014). FIB/SEM technology permitted the full 3D reconstruction of up to 248 spines and their synaptic inputs, thereby allowing us to perform a fine analysis of synaptogenesis in these neurons.

## Materials and methods

### Retroviral tracing

We used a CAG-GFP retrovirus (RV) stock encoding for GFP (Zhao et al., 2006) (a generous gift from Fred H. Gage, Salk Institute, CA, USA). To visualize PSD-95 clusters in newborn granule cells, we used the retroviral vector MRSVPSD95g (Kelsch et al., 2008). RVs were produced by transient transfection of 293 cells as described previously (Zhao et al., 2006). RV stocks were concentrated to working titers of 1 × 10^7^−2 × 10^8^ pfu/ml by means of ultracentrifugation. Adult mice of either sex (7–8 weeks old) were anesthetized and placed in a stereotaxic frame. The scalp was incised, and holes were drilled in the skull. Targets with coordinates (in mm) relative to bregma in the anteroposterior, mediolateral, and dorsoventral planes were as follows: [−2.0, 1.4, 2.2]. 1.5 μl of virus solution per DG was infused at 0.2 μl/min via a glass micropipette.

### Tissue preparation

After 3–4 (*N* = 3 mice) and 8–9 (*N* = 2) weeks, animals were anesthetized by isofluorane inhalation and intracardially perfused with 4% paraformaldehyde and 0.1% glutaraldehyde in 0.12M phosphate buffer (PB). The brain was then extracted from the skull and postfixed overnight in 4% paraformaldehyde. Vibratome slices (~100 μm) were cryoprotected with 30% saccharose in 0.12M PB and permeabilized by three freeze-thawing cycles, immunostained with a rabbit polyclonal anti-GFP antibody (Invitrogen #11122, 1:1000), a biotinylated goat anti-rabbit secondary antibody, and the ABC-peroxidase kit (both from Vector Labs) and developed with DAB and hydrogen peroxide. Slices were postfixed in 2% osmium tetroxide, incubated in 2% uranyl acetate, and flat-embedded in Araldite. All animals were handled in accordance with the guidelines for animal research set out in the European Community Directive 2010/63/EU, and all procedures were approved by the local ethics committee of the Spanish National Research Council (CSIC) and by the Ethics Committee for Animal Experimentation (CEEA), University of Barcelona (Barcelona, Spain).

Araldite-embedded slices containing DAB-labeled cells were glued on the top of araldite blocks and studied under light wide-field microscope (LM). The following three criteria were used to select the dendritic segments to be sampled later by FIB/SEM: (i) they were located in the mid-molecular layer of the dentate gyrus, 50 to 100 μm from the soma, where spines are numerous in adult GC dendrites (except for 3- to 4-week-old GCs, where 6 spines belong to the mid-molecular layer and 20 spines to the inner molecular layer); (ii) the dendritic tree was intensely and homogeneously labeled with DAB; and (iii) they were relatively straight segments that coursed parallel to the surface of the block. Although dendrites coursing in any direction can be sampled, this optimal orientation permits the acquisition of long series of images without the need to displace the field of view of the FIB/SEM microscope during the run.

Once the dendritic segment had been selected, the exposed surface of the block was removed using an ultramicrotome until the selected dendrite was 3 to 5 μm below the surface, so it was readily accessible for imaging by FIB/SEM. We finally acquired optical images of the surface of the final sample.

### Three-dimensional electron microscopy using FIB/SEM technology

Afterwards, the blocks were treated as required to be imaged by the FIB/SEM microscope (Merchan-Perez et al., 2009). They were glued onto a sample stub using a conductive adhesive tab. To avoid charge artifacts, all surfaces of the block except the sample were painted with colloidal silver paint and dried in a vacuum chamber overnight. The blocks were then sputter-coated with gold/palladium for 15 s to facilitate charge dissipation.

**Figure 1:**
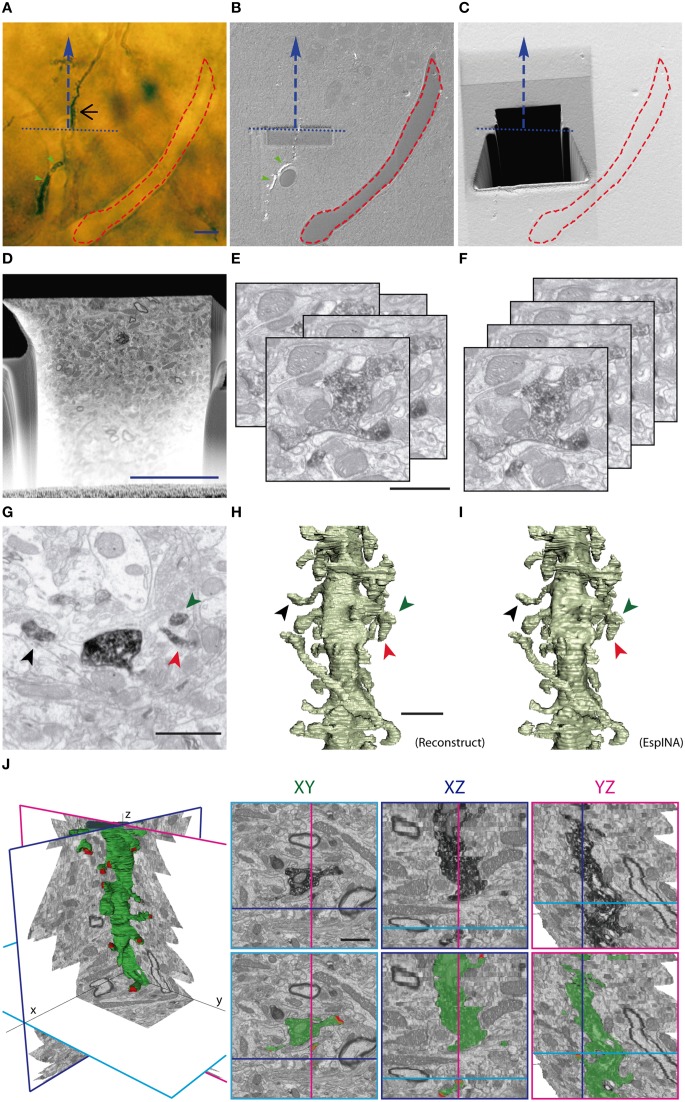
**Correlative light and FIB/SEM microscopy of DAB-stained GC dendrites allows high-resolution 3D reconstruction. (A)** Light microscopy image of the araldite block surface (after trimming) allows the visualization and selection of the DAB-stained dendrite (black arrow) and the annotation of surface fiducial landmarks, such as blood vessels (red dashed line). The course line (blue dashed arrow) defines the trajectory of the dendrite of interest and the selected direction for serial milling and image acquisition. The blue dotted line indicates the desired acquisition starting plane. **(B)** SEM image of the block surface, revealing conserved traits (red dashed line, green arrowheads), allows the identification of the pre-selected starting plane for serial image acquisition. Note that dendritic segments that evolve at the block surface are visible in SEM (green arrowheads), but not dendritic segments evolving entirely below the surface (black arrow in **A**). **(C)** A trapezoidal trench has been milled behind the starting line (blue pointed line) to gain access to the region of interest. Afterwards, a smaller trench has been sequentially milled and imaged in the direction indicated by the blue dashed line. **(D)** Low magnification SEM backscattered electron image showing a freshly milled surface of the trench face during one of the milling-imaging cycles. The dendrite of interest is labeled by a red arrow. **(E,F)** Image acquisition provides up to several hundred serial images **(E)** that require alignment procedures **(F)** to obtain properly oriented stacks ready for 3D visualization and segmentation. **(G,H)** Spine identification in individual micrographs **(G)**; stacks serial images can be further traced to obtain 3D reconstructions performed with either manual segmentation with Reconstruct software **(H)** or with the EspINA software, which allows faster semi-automated reconstructions **(I)**. Note that the overall quality of 3D reconstructions using Reconstruct or EspINA are similar. **(J)** EspINA software allows the segmentation and visualization of labeled structures on the three orthogonal planes before and after segmentation (upper and lower rows, respectively). Scale bars are 10 μm in **(A,D)** 1 μm in **(E,J)**.

3D brain tissue samples were obtained using an electron microscope that combines a focused ion beam (FIB) and a high-resolution field emission scanning electron microscope (SEM) (Crossbeam® Neon40 EsB, Carl Zeiss NTS GmbH, Oberkochen, Germany). This instrument uses a focused gallium ion beam that can mill the sample surface, removing thin layers of material on a nanometer scale. The samples were introduced in the SEM column, and low magnification images of the whole surface of the block were acquired with the secondary electron detector of the column (Figure 1B).

In order to accurately locate the selected dendritic segment for FIB/SEM image acquisition, we used the pair of OM and SEM microphotographs that were taken from the same tissue block. These two images were matched and overlaid using Photoshop (Adobe Systems). The block borders, surface impurities, and exposed DAB precipitates were visible in both microphotographs, so they were used as landmarks to correctly superpose the two images. As a result, we were able to trace the exact position of the selected dendritic segment (only visible in LM images) on the SEM microphotograph (Figures 1A,B).

The sample was then precisely oriented inside the column so as the viewing direction matched the preferred direction of the dendritic segment. A first coarse cross-section was milled with the FIB with a 10 nA gallium beam as a viewing channel for SEM observation at the appropriate location (Figure 1C). Exploration of the exposed surface helped to identify the target dendrite and to choose the final framing. Next, fine milling of the exposed surface was performed with the FIB, using a beam current of 750 pA, which removed a thin layer of material. After removing each slice, milling was paused, and the freshly exposed surface was imaged with a 1.7 kV acceleration potential using the in-column energy-selective backscattered electron detector. Imaging current was 1.2 nA; pixel dwell time was 100 ns and line averaging was set to four. Milling and imaging were sequentially repeated and long series of images were acquired through a fully automated procedure, thus obtaining a stack of images that represented a 3D sample of the tissue (Merchan-Perez et al., 2009). Image resolution on the XY plane was set to 3.7 nm/pixel. Resolution on the Z axis—equivalent to the thickness of the layer of material removed by the FIB in each cycle—was 25 nm. We found that 2048 × 1536 pixel serial micrographs (field of view of 7.6 × 5.7 μm, equivalent to 15000x magnification) allowed unambiguous identification of synaptic components and scanning cycles of about 3 min per microphotograph. For instance, in our study, the largest sample used—comprising 442 serial images—was obtained in a single overnight session of about 22 h, with little or no supervision. We therefore selected these values as the routine settings for obtaining image stacks for 3D reconstructions.

Automatic alignment (rigid registration without rotation) of the stacks of images and signal normalization across slices was performed with Fiji (Schindelin et al., 2012), and 3D reconstruction of the labeled dendritic segments and synaptic contacts was carried out using an improved version of the software packages Reconstruct (Fiala, 2005) and EspINA (Morales et al., 2011) (freely available at http://cajalbbp.cesvima.upm.es/espina/). Exploratory navigation through the stacks of images was performed either with Fiji or EspINA. Binary segmentations of dendrites and synapses were next used to generate surfaces using Imaris software.

A total of 7 mice were processed (1 for the 3-week-old group, 2 for the 4-week-old group, and 3 for the 8-9-week-old group), and 2 to 6 acquisitions were obtained from each group. Each acquisition comprised a tissue volume of between 67 and 481 μm^3^ (mean 237 μm^3^) that included at least one labeled dendritic segment.

### Analysis of afferent bouton connectivity

Various spine protrusions were identified and catalogued in a database, and information related to spine morphology, synapse presence and location, and innervating bouton connectivity were carefully annotated and reviewed by at least three independent specialized scientists. To assess spine morphology classification, criteria was based on current classifications (Harris et al., 1992; Rochefort and Konnerth, 2012). We ended up with five spine types: thin (spines with small necks tipped by small round heads), filopodial (thin and long spines with a pointed PSD, with similar diameters in the neck and head), stubby (thick and short spines with no size differences between neck and head and spine length similar to neck width), mushroom (spines tipped by large heads typically displaying U-shapes), and branched (spines with more than 1 head arising from a single neck). Axon terminals presynaptic to the labeled spines were similarly reconstructed; the number and location of synapses and postsynaptic elements were recorded.

### Image segmentation and quantitative morphometric analysis

3D reconstruction of the labeled dendritic segments and synaptic contacts was carried out with EspINA software (Morales et al., 2011). Briefly, aligned and normalized stacks were further processed with a Gaussian blur filter with a 10-pixel radius. The former “clean” stack was used for user-based segmentation, whereas the “blurred” stack served for automatic, seed-based segmentation in the same work environment. By combining both features, DAB-labeled dendrites and their spines were completely segmented along the stack. Furthermore, their synaptic specializations were segmented by manually tracing closed contours around both the PSD and the apposed presynaptic membrane in consecutive microphotographs. Each segmented synaptic junction was identified independently. We exported the image segmentation binary files into the Imaris platform (Bitplane). Using this software, we generated 3D objects that mimicked the segmentations by an absolute intensity and maximal thresholding approach, without any smoothing step. This allowed for a completely reproducible algorithm of 3D object generation, devoid of any user-biased subjective thresholding step. Next, all spines were cut from their parent dendritic shaft through the base of their neck in a 3D optimal orientation. Branched spines were duplicated and saved in different files to be analyzed separately, and further cut into individual spines at their shared neck isthmus. The volume and sphericity of the final 3D objects generated were annotated. Using Imaris, we calculated the following parameters: dendritic spine volume; synapse size (defined as the volume containing both the postsynaptic density and the presynaptic apposed membrane); and spine and synapse sphericities (defined as the ratio of the surface area of a sphere to the surface area of the structure analyzed, both having the same volume). The sphericity value provides a quantitative record of the morphological complexity of the 3D-reconstructed spines and synapses, since spherical objects would yield a sphericity value of 1, while more complex shapes with larger surface-to-volume ratios would yield progressively lower values (Wadell, 1935). In practice, the surface of reconstructed objects will not be smooth due to the faces and edges of voxels. However, this effect will equally affect all our reconstructions, since voxel size has been kept constant for all of them. Thus, the possible distortions of sphericity measurements will be similar in all reconstructions, and the comparison between them will still be valid. Next, all spines were cut from their parent dendritic shaft through the base of their neck in a 3D optimal orientation. Branched spines were duplicated and saved apart for separate analysis, and further cut into individual spines by their shared neck isthmus. The volume and sphericity of the final surfaces generated were annotated. We calculated the following parameters: dendritic spine volume, synapse size (defined as the volume containing both the postsynaptic density and the presynaptic apposed membrane), and spine and synapse sphericities (defined as the ratio of the surface area of a sphere to the surface area of the structure analyzed). For paired analysis of parameters of spine-synapse couples, a database was generated that included each spine and synapse-paired identifiers, as well as the morphometric values (volume and sphericity) associated with each item. Spines analyzed corresponded to fully 3D-reconstructed individualized spines from GCs aged 8–9 weeks. Correlation was statistically analyzed by non-parametric, two-tailed Spearman test. Binned analysis in the 8–9 week GCs was performed by further pooling and averaging of these data inside bins of constant width. We chose optimal bin widths of spine volume that allowed both the maximal number of values per bin while giving a maximal number of bins in the different analyses. Bins including a single data point were excluded. The bin width used was 5.0E + 06 nm^3^ for all analyses, including spine volume–synapse size (*n* = 21 bins; *n*' = 16 bins in the lower range), spine volume–spine sphericity (*n* = 21; *n*' = 6) and spine volume–synapse sphericity (*n* = 21; *n*' = 12). Linear regressions were performed by best-fit approaches and were statistically tested to be different from zero with the statistical software GraphPad Prism (GraphPad Software). Thresholds were determined by optimizing the goodness of fit (R^2^) of these regressions in the data points inside the lower range. Comparisons of these parameters between two experimental groups were assessed by the non-parametric Mann–Whitney test.

## Results

### FIB/SEM allows the analysis and high-resolution 3D reconstruction of synaptic interactions from identified neurons

To map the onset and development of synaptic inputs on adult-generated GCs in the DG, young adult mice were injected with a retroviral vector (MRSVPSD95g, (Kelsch et al., 2008)) expressing the postsynaptic protein PSD95 fused to GFP, a procedure that allows the visualization of postsynaptic densities (PSDs). While spines were rare in 2-week-old GCs, 3- to 4-week-old neurons displayed numerous spines, most of them tipped with PSD95-GFP-positive puncta (Supplementary Figure 1). Spines and PSD95-GFP-positive PSDs were more abundant at 8–9 weeks, when synaptogenesis is believed to be completed. These findings are consistent with previous studies on adult neurogenesis in the DG (Toni et al., 2007; Toni and Sultan, 2011) and prompted us to focus our FIB/SEM analysis on dendrites of adult-generated GCs aged 3–4 and 8–9 weeks.

**Figure 2:**
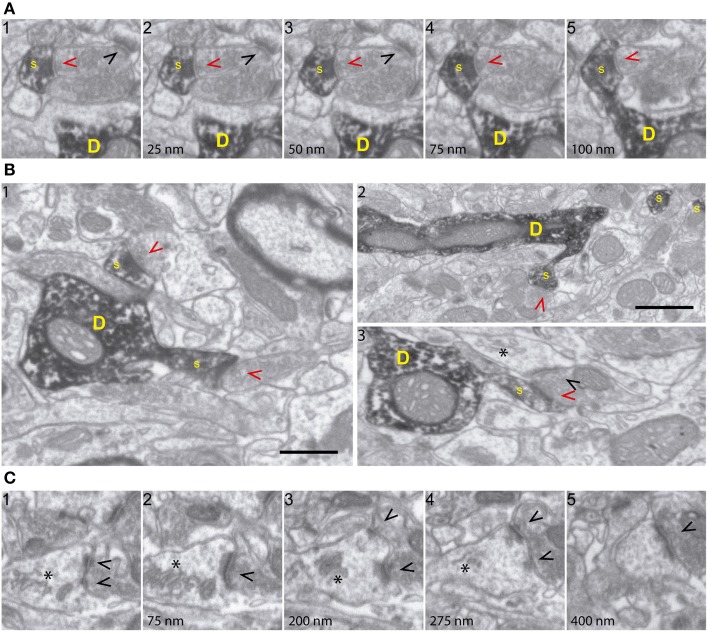
**FIB/SEM microscopy allows high-resolution ultrastructural analysis of identified synapses. (A)** Five consecutive serial images (a1-5; spaced 25 nm each) demonstrating high fine structural resolution of GFP/DAB-stained dendrites, on both the XY and Z axes. The sequence shows a spine (s) emerging from the parent dendrite (D) and a presynaptic terminal forming a synapse with the labeled spine (red arrowhead) and with an unlabeled spine (black arrowhead). Note that 25 nm thick Z-axis image acquisitions allow efficient and repetitive visualization of structures of interest, such as synapses and spine necks. **(B)** Various FIB/SEM images (b1-3) demonstrating overall ultrastructural quality and the unambiguous identification of dendrites (D), spines (s), and axon terminals establishing synapses with either labeled (red arrowheads) or unlabeled (black arrowheads) profiles. **(C)** Selected serial/correlative images (c1-5; spaced 75–125 nm) showing distinct features, including spine apparatus (asterisk) and a perforated synapse (black arrowheads), on a single unlabeled spine. Scale bar in **(B1)** is 0.5 μm and applies to all panels, except for **(B2)**, which corresponds to 1 μm.

To address the development of synaptic inputs with EM resolution, adult-generated neurons were labeled with retroviral vectors expressing GFP. Brain slices were processed for GFP-immunostaining, diaminobenzidine (DAB) development, and plastic embedding using conventional TEM procedures. Flat embedding of slices allowed the identification of labeled GCs and the subsequent trimming of tissue blocks. We next designed a correlation procedure that allowed us to apply FIB/SEM technology to identified dendrites previously selected under the light microscope (LM) (Figure 1A). In brief, labeled and straight dendritic segments evolving parallel to surface were identified and their precise position annotated with reference to fiducial landmarks present in both the LM and EM images (Figures 1B,C). Examination of these images revealed an overall quality of fine structure and resolution comparable to that of conventional TEM (Figures 1, 2).

As a further step for the automation and analysis of 3D reconstructions, we implemented the EspINA program by developing specific software for the reconstruction of labeled structures. Alignment of images, segmentation, and 3D reconstructions of tissue blocks of up to 10 μm in depth were efficiently obtained in a semi-automatic manner: alignment of FIB/SEM images was assessed by automatic registration with FIJI (Schindelin et al., 2012) and manually checked with Reconstruct software (Fiala, 2005). The resulting images were saved as stacks, and connectivity parameters were analyzed and annotated by visualizing them with FIJI (Figures 1D–G). Examples of 3D dendritic segments reconstructed using EspINA are shown in Figure 1I and were equivalent to segments reconstructed using the standard Reconstruct software (Figure 1H). Although EspINA-based reconstruction still requires frequent user intervention, in our experience it is at least 25% faster than fully manual reconstruction.

Moreover, EspINA-based 3D reconstructions allowed quality control by the researcher and the visualization of orthogonal sections on any of the XYZ axes (Figure 1J). Even when automatic or semiautomatic 3D reconstructions were not possible, manual reconstructions were facilitated by a good resolution in the z axis (25 nm in our study), and by the fact that images were virtually free of deformation artifacts, which allowed almost perfect alignment of serial images.

**Figure 3:**
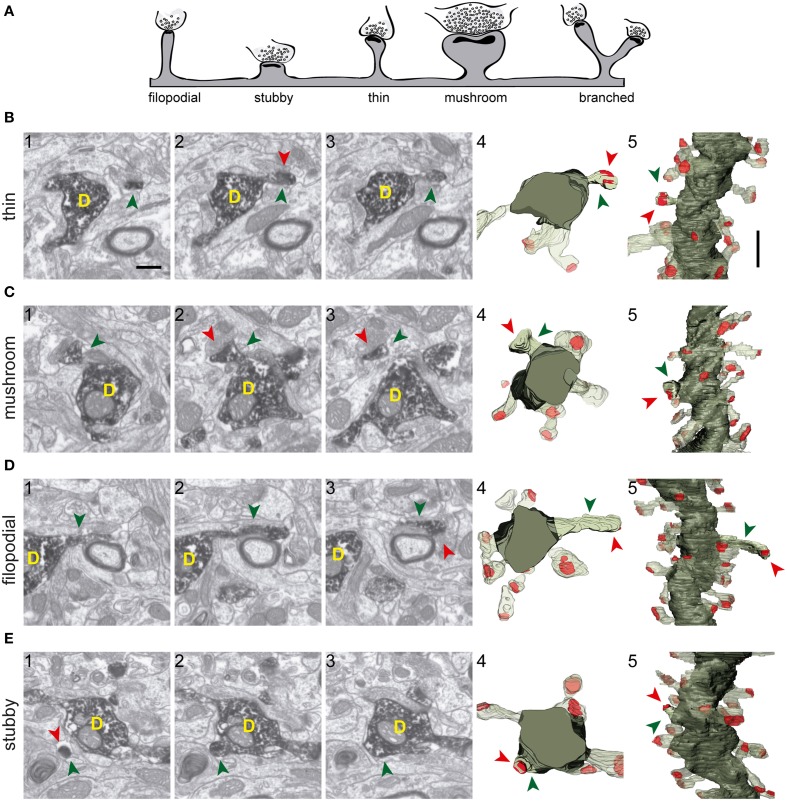
**Types of spines arising from 8-week-old GFP/DAB-labeled GCs as reconstructed with FIB/SEM microscopy. (A)** Schematic representation of four e types of spines defined in the present study. Examples of thin **(B)**, mushroom **(C)**, filopodial **(D)**, and stubby **(E)** spines arising from their parent dendrite (D). The left images (1–3) show selected serial planes of the spines depicting the head (green arrowheads), neck, and synaptic contact (red arrowheads). The right 3D reconstructions (4–5) show the labeled spines in two orthogonal orientations. The dendritic shaft (D) is shown in solid dark green, the spine of interest in solid pale green, and its synapse in solid red. Neighboring spines and synapses are indicated in light pale green and red, respectively. Scale bar in (**B1**) is 0.5 μm and applies to **(B–E 1–4)**. Scale bar in **(B5)** is 1 μm and applies to **(B–E5)**.

**Figure 4:**
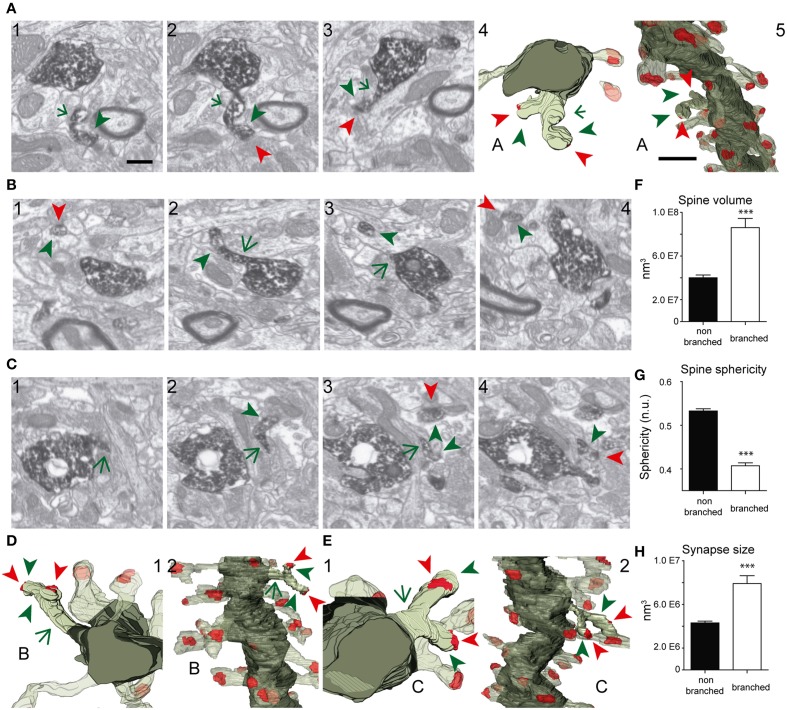
**FIB/SEM images and the corresponding 3D reconstructions illustrating branched spines in GCs aged 8–9 weeks. (A–C)** Serial FIB/SEM images illustrating three examples of branched spines: **A1–3** (spine A), **B1–4** (spine B), and **C1–4** (spine C). The corresponding 3D reconstructions are shown in two orthogonal orientations in panels **A4,5** (spine A), **D1,2** (spine B), and **E1,2** (spine C). The labeling of synaptic contacts is as in Figure 3. The spine heads are shown by green arrowheads, the shared neck by a green arrow, and their synaptic contacts by red arrowheads. The colors in the 3D reconstructions are as follows: the dendritic shaft in solid dark green, the spine of interest in solid pale green, and its synapses in solid red. Neighboring spines and synapses are colored in light pale green and red, respectively. **(F–H)** Histograms showing average spine volume **(F)**, spine sphericity **(G)**, and synapse size **(H)** in non-branched and branched spines. Data represent mean ± SEM; ^*^p < 0.05; ^**^p < 0.01; ^***^p < 0.001; Mann–Whitney test. Scale bar in **(A1)** is 0.5 μm and is applicable to **(A–C 1–4, and D-E1)**. Scale bar in **(A5)** is 1 μm and is applicable to **(A5, D2, and E2)**. Abbreviations: *n.u*., no units.

Qualitative analyses of 3D reconstructions allowed us to trace identified spines back to the parent dendrites and to study the 3D architecture of synaptic interactions and the fine structural features of synapses and presynaptic elements (**Supplementary Movies 1, 2**, Figures 1–**4** and Supplementary Figure 2). Thus, cell membranes, cytoskeletal components, and organelles were clearly identifiable. Hence, DAB-labeled dendrites and the spines arising from them were recognizable, as were the unlabeled presynaptic boutons filled with synaptic vesicles and establishing synaptic contacts with DAB-traced profiles (Figures 2A,B). PSDs and organelles present in axonal and postsynaptic (GFP-labeled) elements were clearly identifiable, including spine apparatus arranged in stacks, ER cisternae, and mitochondria (Figure 2C). We conclude that FIB/SEM technology is a reliable and straightforward procedure that allows high throughput, high resolution, semi-automated 3D analyses of identified neuron-to-neuron synaptic interactions at the ultrastructural level.

### Three-dimensional analysis of input synapses onto mature adult-generated granule cells

We first focused on neurons aged 8–9 weeks, when adult-generated GCs are considered to reach maturity (Zhao et al., 2006). Six dendritic segments were analyzed, allowing the 3D reconstruction of up to 271 spines, of which 226 were fully reconstructed (**Supplementary Movie 3**; Supplementary Table 1). A qualitative evaluation revealed that most spines were contacted by a single presynaptic bouton. A small percentage, however, were found to lack synaptic contacts (non-synaptic spines, ~2%, *N* = 5) (Supplementary Figure 2A), with all the remaining spines bearing exclusively asymmetric synaptic contacts. Most synapses were established on the spine heads, while ~3% (*N* = 7) received synaptic input on the spine neck (Supplementary Figure 2B). Three spines (~1%) received both an excitatory contact on the spine head and a second synapse on the neck, established by different boutons (not shown).

The shapes and sizes of spines were highly variable. We observed extremely large spines (1.8E8 nm^3^, around 0.60 μm in diameter) and spines with small heads (1.7E6 nm^3^, around 0.25 μm in diameter). 3D reconstructions allowed us to classify spines into 5 main types: thin, filopodial, stubby, mushroom, and branched (Figure 3) (Peters and Kaiserman-Abramof, 1970; Harris et al., 1992; Bourne and Harris, 2008). The largest proportion of spines corresponded to the thin and mushroom categories (43 and 20%, respectively). Lower percentages were found for the filopodial and stubby categories (17 and 5%, respectively) (Supplementary Table 1). Furthermore, up to a 15% of the spines were branched. In general, such complex spines had two side branches (Figures 4A–E); however, we also found spines displaying up to three distinct tips. Virtually all the extensions that arose from these branched spines were tipped by synapses, which were established by various presynaptic terminals, thereby indicating that these spines were poly-innervated (**Supplementary Movies 4, 5**). We also classified the single spine heads present in branched spines. Interestingly, the percentage of spine types (filopodial, thin, mushroom, and stubby) in branched spines was similar to that of the whole population of spines (**Figure 6B**), indicating both individual heterogeneity in branched spines and robust conservation of spine categories. To our knowledge this is the first study reporting ramified, branched spines in adult-generated GCs. We compared the morphological parameters between both types of spine. Overall spine and synapse sizes were markedly larger in branched spines, which showed less sphericity, thus reflecting their complexity (Figures 4F–H).

**Figure 5:**
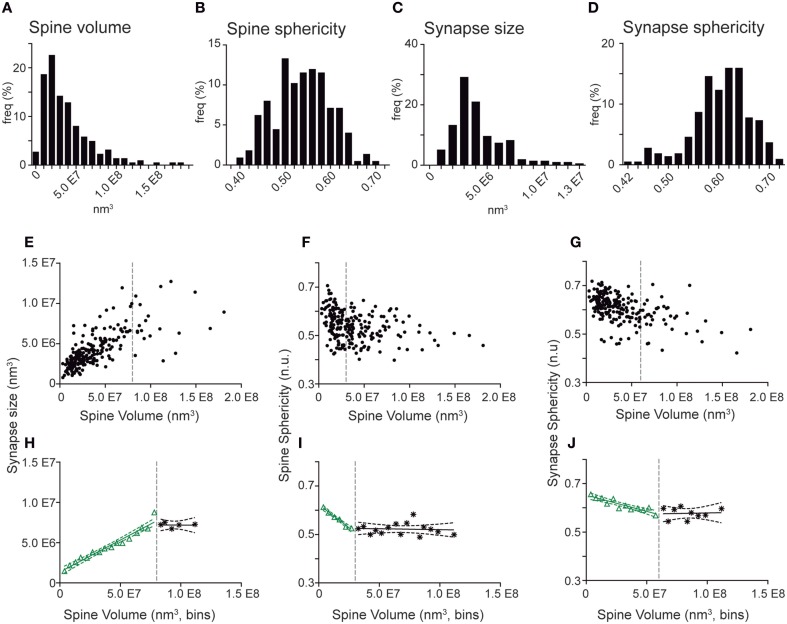
**Quantitative correlations of spine volumes and other morphometric parameters in spines and synapses from GC aged 8–9 weeks. (A–D)** Frequency histograms show the distribution of spine volume **(A)**, spine sphericity **(B)**, synapse size **(C)**, and synapse sphericity **(D)**. Note that all distributions display a continuous range of values. **(E–G)** Plots showing correlation of individual spine volumes with synapse size **(E)**, spine sphericity **(F)**, and synapse sphericity **(G)**. **(H–J)**. Binned analysis of the data shown in **(E–G)** revealing linear regressions between spine volume and synaptic size **(H)**, spine sphericity **(I)**, and synapse sphericity **(J)** above (black) and below (green) defined volume thresholds (dashed gray lines). Dashed green and black lines represent the 95% confidence intervals for these fits. The data show that the three parameters evolve linearly with spine volumes until a certain threshold, after which the three parameters remain constant. Note in **(E–G)** that these parameters no longer correlate above these thresholds. Detailed correlation analyses are provided in Supplementary Table 2.

We next took advantage of the complete 3D reconstructions to analyze the morphometric parameters of the spines (Figure 5). Spine and synapse sizes were distributed with a left-skewed curve, whereas sphericities distributed symmetrically around the means (Figures 5A–D). When spine volumes were correlated with other parameters, we found a positive correlation with synapse sizes (Spearman r 0.7414, *p* < 0.001) and a negative correlation with the spine and synapse sphericities (Spearman r of −0.3566 and -0.5016, *p* < 0.001, respectively; (Supplementary Table 2, Figures 5E–G). To further analyze such distributions, spine volumes were binned, and the pooled points inside each bin were averaged (Figures 5H–J). In all cases, the dependent variable evolved linearly with increasing spine volume until reaching a certain threshold, upon which it appeared to remain constant. These data suggest that above a given spine volume threshold, synapse size and sphericity remain unchanged (Figures 5E–G, Supplementary Table 2).

Taken together, the present FIB/SEM analyses highlight the complex synaptic architecture of spines in mature GCs and allowed us to describe vacant spines and branched spines, as well as to correlate spine and synaptic sizes and sphericity.

### Developmental analysis of input synapses onto adult-generated GCs

**Figure 6:**
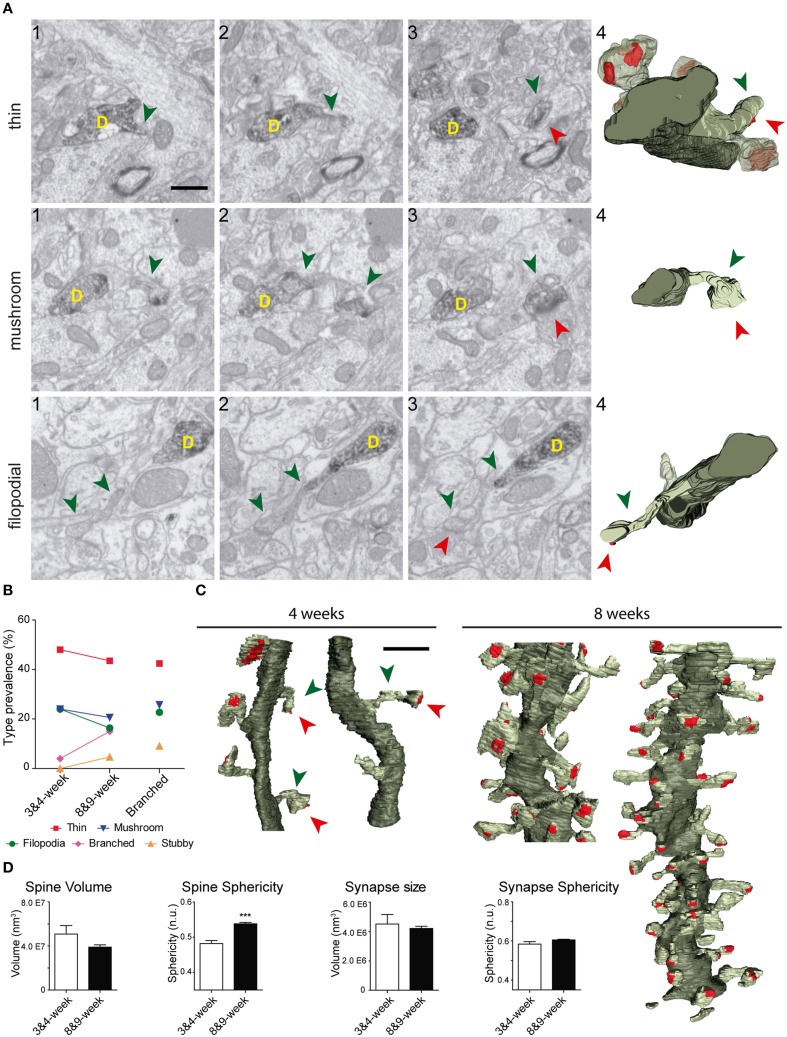
**Comparative analysis of spines in GC aged 3–4 and 8–9 weeks. (A)** Examples of thin, filopodial, and mushroom spines arising from their parent dendrite (D) in 3- to 4-week-old GCs. The three left images (1–3) show selected serial planes of the spines, depicting the head (green arrowheads), neck, and synaptic contact (red arrowheads). The 3D reconstructions are shown to the right (4). **(B)** Plots showing the percentages of the different types of spines at 3–4 and 8–9 weeks; percentages of spine types are also shown for branched spines (right). **(C)** 3D reconstructions allowing comparison of dendritic segments and spines at 3–4 and 8–9 weeks. The color code is the same as described in Figure 3. **(D)** Histograms showing spine volumes and sphericity and synapse size and sphericity at both ages. Data represent mean ± SEM. ^***^p < 0.001; Mann–Whitney test. Scale bar in **(A)** is 0.5 μm. Scale bar in **(C)** is 1 μm.

To study the development of dendritic spines in adult generated GCs, we performed 3D reconstructions of these structures in neurons aged 3–4 weeks. We found eight spines in two dendritic segments of 3-week-old GCs, and 20 spines in six segments of 4-week-old GCs, of which 22 were fully reconstructed (Figure 6). As illustrated by our 3D reconstructions, the overall shapes of dendritic spines at 3–4 weeks were similar to those described for 8–9 week-old GCs (Figures 6A–C). To characterize developing GC spines, we pooled data from 3- and 4-week-old neurons (Supplementary Table 1). We did not find non-synaptic spines at these ages, and all synaptic contacts were on the spine heads. The vast majority of spines bore a single synapse, but we found two spines (~7%) receiving more than one synaptic contact on their heads (from different boutons), a feature not found in mature GCs. Regarding the shapes of the spines, 48% were thin, 24% mushroom, and 24% filopodial. We also found one branched spine (4%) with three tips, but stubby spines were not found in GCs aged 3–4 weeks (Figure 6B, Supplementary Table 1).

A comparison of spine types at 3–4 and 8-9 weeks revealed similar percentages of asynaptic, thin, and mushroom categories at both ages, and slightly less filopodial spines at 3–4 weeks (Figure 6B). Moreover, in addition to the lack of stubby spines, branched spines were underrepresented at 3–4 weeks. These data show that while thin, mushroom, and filopodial types are constant, ramified and stubby types are a predominant feature of mature GCs.

We also observed that developing spine volumes correlated positively with synaptic sizes (Spearman r 0.8060, *p* < 0.001) and negatively with spine sphericity (Spearman r −0.6718, *p* < 0.01) (Supplementary Figure 3). When compared to 8–9 week-old GCs, spines at 3–4 weeks were less spherical and tended to be larger (Figure 6D). Taken together, our data show that although there is a remarkable robustness in most morphological and morphometric parameters at both ages, stubby and branched spines are clearly a characteristic feature of mature GCs, and spines decrease in size and complexity with age.

### Spines from adult-generated GCs are preferentially innervated by multi-synaptic axon terminals

**Figure 7:**
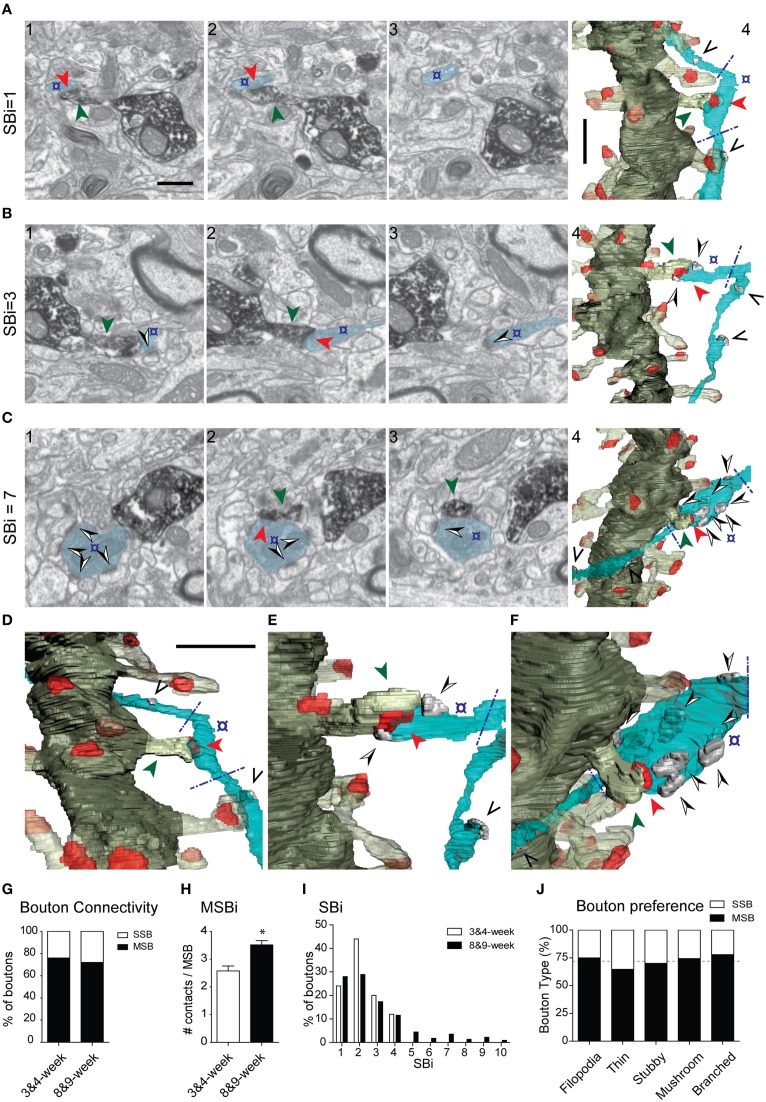
**Presynaptic innervation of GC spines at 3–4 and 8–9 weeks. (A–C)** Three examples of synaptic configurations. The left FIB/SEM images (1–3) show selected serial planes of the dendritic spines and presynaptic boutons; **(A)** presynaptic bouton (o) contacting (red arrowhead) exclusively the DAB-labeled spine (green arrowhead); **(B,C)** axon terminals forming complex synaptic configurations contacting both the labeled spine and several unlabeled dendritic spines (black and white arrowheads) **(B,C)**. The corresponding 3D reconstructions are shown to the right (**A4**, **B4**, **C4**), as well as magnified tilted orientations in **D**–**F**, respectively. The number of postsynaptic spines innervated by the same bouton (SBi, Synaptic Bouton index) is shown to the left. Note that only the varicosities presynaptic to the labeled spine were analyzed (delimited by blue dashed lines in the 3D panels). The axons may establish other synapses elsewhere, not analyzed (black arrowheads in the 3D panels). The example shown in **(B)** illustrates a multisynaptic bouton establishing a total of three synapses and the example illustrated in **(C)** establishes seven synapses. The color code is as described in Figure 3; additionally, the axon is shown in light blue, and synapses established by the axon onto non-labeled spines in solid gray. **(G)** Percentage of single-synaptic (SSB) and multi-synaptic (MSB) boutons in dendritic spines aged 3–4 and 8–9 weeks. **(H)** Average number of synaptic contacts established by MSBs at 3–4 and 8–9 weeks. **(I)** Histogram showing the frequency of synaptic contacts established by axon terminals at 3–4 and 8–9 weeks.** (J)** Multisynaptic boutons innervate all spine types and morphologies equally. Percentage of single-synaptic (SSB) and multi-synaptic (MSB) boutons in various types of dendritic spines in 8- to 9-week-old neurons; the dashed line indicates the overall percentage of SSBs and MSBs. Data represent mean ± SEM. ^*^p < 0.05; Mann–Whitney test. Scale bar in (**A1**) is 0.5 μm and applies to **(A–C, 1–3)**. Scale bar in **(A4)** is 1 μm and applies to **(A–C4)**. Scale bar in **(D)** is 1 μm and applies to **(D–F)**.

We next examined axon terminals that were presynaptic to labeled GCs. We analyzed the connectivity of 271 terminals innervating identified spines (Figure 7). At 8–9 weeks, about one fourth (28%) of presynaptic boutons established synapses exclusively onto the GFP-labeled spine (Single Synaptic Boutons, SSBs; Figures 7A–D). The remaining axon terminals (72%) formed synapses with both the labeled spine and with one or more additional postsynaptic elements, the majority of these also being spines (Multiple Synaptic Boutons, MSBs). All the synapses were asymmetric. Most MSBs established a synapse with one to three unlabeled spines, in addition to the GFP-positive spine (Figures 7B,E). Interestingly, up to 26% of axon terminals were involved in complex synaptic configurations, establishing simultaneous synapses with four or more spines, in addition to the identified spine (Figure 7I). Some MSBs (8%) exhibited highly complex configurations and established synapses with 7–10 postsynaptic elements (Figures 7C,F, **Supplementary Movies 6, 7**). Finally, the SSB/MSB ratio was similar for all spine types (Figure 7J), and spines postsynaptic to either SSBs or MSBs did not differ in their morphometric properties in neurons aged 8–9 weeks (spine volume: 0.036 ± 0.027μm^3^, 0.040 ± 0.027 μm^3^, respectively; spine sphericity: 0.543 ± 0.008, 0.535 ± 0.005, respectively; synapse size: 4.07E + 6 ± 2.55E + 5 nm^3^, 4.24E + 6 ± 1.85E + 6 nm^3^, respectively; synapse sphericity: 0.60 ± 7.66E-3, 0.61 ± 4.56E-3, respectively. No significant differences were found; Mann–Whitney test).

At 3–4 weeks, six out of 25 axon terminals (24%) established a single synapse exclusively with the GFP-labeled spine, whereas 19 terminals (76%) established contacts with more than one postsynaptic element (11 of them with one additional element, and 8 boutons with 2–3 unlabeled spines, in addition to the GFP-traced spine) (Figure 7G and Supplementary Table 1). The mean number of contacts established by MSBs was higher at 8–9 weeks (Figure 7H), since terminals establishing synapses with five or more spines were not found at 3–4 weeks (Figure 7I). Thus, while the percentage of MSBs was similar at 3–4 and 8–9 weeks (76 and 72%, respectively), the average number of synapses established by these boutons increased at 8–9 weeks (Figures 7G–I). We conclude that although the innervation of GC spines by MSBs is a common feature of developing and adult spines, the complexity of synaptic multi-innervation increases in mature GCs.

## Discussion

Here we show that the connectivity of newly generated neurons can be studied using FIB/SEM technology, which allows unambiguous identification and 3D analysis of synapses from identified neurons. Only recently, researchers have exploited the potential of FIB/SEM technology to study biological material, including neural tissue (Knott et al., 2008; Merchan-Perez et al., 2009, 2014; Briggman and Bock, 2012; Blazquez-Llorca et al., 2013; Helmstaedter, 2013). However, the complex 3D organization of nervous tissue requires pre-labeling of axons and dendrites from defined neurons. Here we have optimized a feasible and user-friendly procedure to capture FIB/SEM images from single GFP-immunostained (and DAB-processed) neurons.

An advantage of FIB/SEM microscopy is that serial images are obtained in a fully automated manner, with little user interaction once milling and imaging have been programmed, allowing the acquisition of long series of images from the regions of interest. This is a critical advantage of automated EM techniques. For example, in a previous study of the synaptic inputs of identified spines, we were able to reconstruct 144 spines using conventional TEM (Arellano et al., 2007). However, it took us over 2 years to complete. This is because serial-section TEM is susceptible to some important problems, including loss of sections, uneven section thickness, frequent presence of debris or artifacts in sections (e.g. folds) and geometrical distortions. Thus, many spines had to be discarded because they were incompletely reconstructed. All these problems are overcome by using current FIB/SEM technology.

Furthermore, the resulting resolution on the X-Y plane was comparable to that of TEM, since a resolution of around 4 nm/pixel was easily attained. The resolution on the Z axis, in our case 25 nm, proved even better than that of TEM, where uniform serial sections below 60 nm are difficult to obtain. FIB/SEM technology is also free of most of the main artifacts of TEM, such as the loss or folding of sections. Moreover, given that the images are taken from the block face, they are almost completely aligned, and the definitive alignment can also be automated (Merchan-Perez et al., 2009). Thus, the resolution and quality of the images obtained herein were comparable to those obtained with conventional TEM but without the need of manual serial sectioning and with none of the artifacts common to TEM sections.

Another advantage of FIB/SEM technology is the feasibility and accuracy of 3D EM reconstructions. The automated and sequential milling/image acquisition procedure greatly facilitates the harvesting of single images, and the feasibility of the method allows 3D reconstructions of samples up to 10 um thick. The generation and visualization of these 3D reconstructions can be performed in a user-friendly format by means of the EspINA software. For instance, our FIB/SEM approach allowed the identification of rare and unconventional dendritic spines, including extremely thin (filopodial) spines, non-synaptic and branched spines, and complex MSBs.

Finally, as the procedure described here uses standard protocols for TEM, and given the wide use of DAB for the characterization of neurons and their synaptic connections, the FIB/SEM technology developed would be of immediate use for the analysis of conventional TEM samples that have already been prepared. In conclusion, the high resolution, feasibility, and automation of the FIB/SEM technology described make this methodology a technological breakthrough not only for the imaging of identified neural microcircuits using neuron-specific markers, but also for the discovery of features that may have been overlooked.

Hippocampal adult neurogenesis is essential for cognitive processes (Zhao et al., 2008; Deng et al., 2010). Essential issues to tackle include how these new neurons become functionally integrated into pre-existing adult circuits and the identification of the factors that influence this process (Van Praag et al., 2002; Toni et al., 2007, 2008). Previous studies have described the developmental pattern of synapse formation and the establishment of efferent connections by these neurons (Zhao et al., 2006; Ge et al., 2007; Toni et al., 2007, 2008; Sun et al., 2013). Further, the functional integration of these neurons is modulated by a number of factors, including spatial memory training, stimulation of the entorhinal pathway, and the Reelin pathways (Kee et al., 2007; Garthe et al., 2009; Gu et al., 2012; Teixeira et al., 2012). However, how this integration takes place and the developmental modifications that occur during this process remain largely unknown. Here, we applied FIB/SEM technology to characterize mature synaptic inputs onto adult-born GCs. Although our observations largely support previous conventional TEM studies (Toni et al., 2007, 2008), several interesting features were revealed. Complex branched spines displaying up to four individual protrusions and receiving independent synaptic inputs accounted for up to ~15% of the spines. Although previous TEM studies pointed to the presence of branched spines in the DG (Geinisman et al., 1989; Trommald et al., 1996; Trommald and Hulleberg, 1997; Popov and Stewart, 2009), our study represents the first description of this type of spine in adult-generated GCs. Given current views on the relevance of the shape of spines for their physiological and integrative properties, it is likely that such complex ramified spines have a physiological impact on the dendritic physiology of adult-generated GCs (Rusakov et al., 1996; Yuste and Majewska, 2001; Harris and Weinberg, 2012; Rochefort and Konnerth, 2012).

The use of serial sections and the narrow spacing between consecutive EM images (25 nm) greatly facilitated the classification of spines into morphological types, since the structure of each spine could be easily compared across several planes and examined as a whole. It must be noted, however, that this classification is only descriptive and used for simplicity given that it is based on qualitative criteria and there is a continuum of spine morphological types (e.g., see Arellano et al., 2007). Nevertheless, this classification is a useful descriptive tool to compare our results with previous studies. For example, we found a considerable number of filopodial-like spines (17%) in mature GCs, while these spines have been traditionally associated with young neurons and immature spines, often lacking postsynaptic specializations (Ziv and Smith, 1996; Konur and Yuste, 2004; Knott et al., 2006; Yasumatsu et al., 2008). However, our data show that virtually all filopodial spines displayed synapses. Conversely, our 3D analyses revealed a low percentage of spines lacking synapses in these mature neurons. All together, our findings indicate that filopodial, branched, and vacant spines are constitutive of adult-generated GC dendrites, probably representing synaptic remodeling intermediate stages in these neurons (Toni et al., 2007; Ge et al., 2008; Toni and Sultan, 2011).

Our study also allowed a morphometric characterization of GC dendritic spines and synapses. This characterization was based on quantitative measurements of spine and synapse volume and sphericity. Furthermore, this quantitative analysis was performed independently of the qualitative classification of spine types. One striking finding is the increase in spine sphericity in mature spines, when compared to young spines (Figure 6). This process has already been described in other neurons and is likely to reflect spine maturation (Knott et al., 2006; Honkura et al., 2008; Racz and Weinberg, 2013). Another finding is that spine volumes correlated with synaptic sizes and with spine and synapse sphericities up to a given threshold (Figure 5), above which both the synaptic size and the spine and synapse sphericities remained constant. To our knowledge, such a two-regime distribution has not been reported previously. The boundaries detected may point to physiological thresholds relevant in the development of spine structural plasticity, and therefore they might be potentially related to calcium and cytoskeletal spine dynamics, among other mechanisms. Furthermore, our results offer a strong ground truth for the study and interpretation of how structural plasticity molds the synaptic elements during the integration of newborn GCs in the preexisting circuitry.

Our comparative 3D analyses on neurons aged 3–4 and 8–9 weeks allowed us to define the synaptogenesis in adult-generated GCs. The percentage of filopodial, thin, and mushroom spines was roughly similar at both ages (though with a tendency to decrease at 8–9 weeks), indicating that these spine types are constitutive of GC dendrites from the onset of synaptogenesis. In contrast, stubby spines were observed exclusively in mature GCs and branched spines were very rare at early stages. Therefore, while filopodial, thin, and mushroom spines appear to play a major role in the special electrophysiological properties of young adult-generated GCs, including hyperexcitability and low LTP threshold (Zhao et al., 2006; Ge et al., 2007), stubby and branched spines may contribute specifically to the physiological properties of mature GCs.

A previous study described that up to 40% of axon terminals that are presynaptic to newborn GCs are simultaneously enrolled in synapses with unlabeled spines (MSBs) (Toni et al., 2007). Our FIB/SEM study confirms this observation and adds two important findings. First, the percentage of MSBs establishing synapses with other targets is substantially higher (72%), and second, we describe the presence of highly complex synaptic configurations in which single boutons simultaneously contact four or more postsynaptic elements, in addition to the GFP-labeled spine (up to 9 additional spines). Although the function of such complex synaptic configurations in GC physiology remains to be elucidated, they have been associated with plasticity and LTP (Toni et al., 1999; Geinisman et al., 2001; Knott et al., 2006). We propose that the activation of a single axon terminal, driving coactive synaptic activity to several GCs, influences the generation of synchronous networks and rhythms in the DG, which are crucial for cognitive processes, including learning and memory (Deng et al., 2010; Aimone et al., 2011; Buzsaki and Moser, 2013). Finally, and although the identity of target spines of MSBs is not known, it is plausible that these complex axon terminals are specialized in driving coactive simultaneous activation to defined GC subpopulations, for instance to the dendrites of newborn GCs.

Our 3D reconstructions revealed that MSBs are equally present in young and mature GCs (about 76% in young GCs), and that the synaptic complexity of the axon terminals contacting GCs clearly increases with maturity (e.g., Figure 7); this finding indicates that such synaptic configurations are a robust feature of GC microcircuits, although the different age-dependent complexities suggest that they may differentially influence the physiological properties of young and adult GCs. Our analysis of two stages of spine development suggests that axons presynaptic to spines arising from immature newborn GCs are more prone to progressively establish additional synaptic contacts.

In summary, here we implemented FIB/SEM technology that allows the 3D analysis of identified, traced neurons, with high resolution and reliability. This technology would be implemental for the characterization of synaptic microcircuits in a high-throughput manner. This technology allowed us to reveal that the synaptic architecture of adult-generated GCs is more complex than previously thought.

### Conflict of interest statement

The authors declare that the research was conducted in the absence of any commercial or financial relationships that could be construed as a potential conflict of interest.

## Supplementary material

Supplementary Figure 1 **Dendritic spines and postsynaptic densities visualized in developing GCs**. Newborn GCs were labeled with a retroviral vector expressing PSD95 and visualized at 3, 4 and 8 weeks post-injection. **(A)** Low magnification views of retrovirally labeled adult-born GCs. **(B)** Confocal reconstructions of GC dendritic segments showing spines (red) and postsynaptic densities (yellow). Note the increase in spines concomitant to post-injection times. Scale bars are 20 μm in **(A)** and 5 μm in **(B)**. Abbreviations: GCL, granule cell layer; ML, molecular layer; 3w, 3 weeks post-infection.

Supplementary Figure 2 **Types of dendritic spines in 8-week-old GFP/DAB-labeled GCs as reconstructed with FIB/SEM microscopy**. Examples show a non-synaptic spine **(A)** and a spine receiving synaptic contact in the neck **(B)**. The images on the left show three selected serial planes (1-3) of the spines depicting the head (green arrowheads), neck, and synaptic contact (red arrowheads), while the images on the right show 3D reconstructions (4,5) of the labeled spines in two different orientations. The dendritic shaft (D) is shown in solid dark green, the spine of interest in solid pale green, and its synapse in solid red. Neighboring spines and synapses are shown in light pale green and red, respectively. Scale bar in **(A1)** is 0.5 μm and applies to **(A1-4, B1-4)**; scale bar in **(A5)** is 1 μm and applies to **(B5)**.

Supplementary Figure 3 **Correlation of 3- to 4-week-old GC spine volume with morphometric parameters**. **(A-c)** Plots showing correlation of individual spine volumes with synapse size (Spearman r=0.8060, p<0.001) **(A)**, spine sphericity (Spearman r=− 0.6718, p<0.01) **(B)**, and synapse sphericity (non-significant correlation, Spearman r= −0.29) **(c)**. Spine volume thresholds observed in the 8-9 week group are illustrated by gray dashed lines.

Supplementary Table 1 **Numbers of analyzed dendritic spines and presynaptic boutons and their classification**.

Supplementary Table 2 **Statistical analysis of correlations between spine and synapse morphometric parameters in 8-9-week-old GCs**.

### Supplementary movie legends:

Entire fileset is available at: http://dx.doi.org/10.6084/m9.figshare.1266450

**Supplementary Movies 1 and 2. Complete 3D image stacks of labeled 8-week-old GC dendrites showing numerous spines, presynaptic boutons, and synapses**. Slice depth respect to the first slice is shown at the lower right corner. Scale bar is 0.5 μm. **Supplementary Movie 1** is available at: http://dx.doi.org/10.6084/m9.figshare.1266442 **Supplementary Movie 2** is available at: http://dx.doi.org/10.6084/m9.figshare.1266443

**Supplementary Movie 3. 3D reconstruction of the dendritic segment displayed in Movie 1**. The dendritic shaft is shown in dark green, the dendritic spines in pale green and their synapses in red. **Supplementary Movie 3** is available at: http://dx.doi.org/10.6084/m9.figshare.1266444

**Supplementary Movies 4 and 5. 3D image stack of an 8-week-old GC dendrite illustrating a branched spine (4)**. Green and red arrowheads show the individual heads and synapses, respectively, and a green arrow shows the shared neck that connects the branched spine with the dendritic shaft (D). Slice depth respect to the first slice is shown at the lower right corner. Scale bar is 0.5 μm. The 3D reconstruction is shown in (5). The dendritic shaft is shown in solid dark green and the branched spine in solid pale green, and its synapses in solid red. Neighboring spines and synapses are indicated in light pale green and red, respectively. **Supplementary Movie 4** is available at: http://dx.doi.org/10.6084/m9.figshare.1266445 **Supplementary Movie 5** is available at: http://dx.doi.org/10.6084/m9.figshare.1266446

**Supplementary Movies 6 and 7. 3D image stack of an 8-week-old GC dendrite illustrating a presynaptic bouton (MSB) forming up to eight synaptic contacts (6)**. A green arrowhead shows the spine head of a GFP-labeled spine receiving a synapse (red arrowhead) from a MSB. Yellow arrowheads show synaptic contacts established by the same MSB with neighboring non-labeled spines. Slice depth respect to the first slice is shown at the lower right corner. Scale bar is 0.5 μm. The 3D reconstruction is shown in (7). The dendritic shaft is shown in solid dark green, the labeled spine contacting the MSB in solid pale green, and its synapse in solid red. Neighboring spines and synapses are indicated in light pale green and red, respectively. The axon that contains the MSB is shown in light blue, and synapses established with non-labeled spines are shown in solid gray. Abbreviations: *MSB*, multisynaptic bouton; *D*, dendritic shaft of the GFP-labeled dendrite. **Supplementary Movie 6** is available at: http://dx.doi.org/10.6084/m9.figshare.1266447 **Supplementary Movie 7** is available at: http://dx.doi.org/10.6084/m9.figshare.1266448
