# Corrigendum: FIB/SEM technology and high-throughput 3D reconstruction of dendritic spines and synapses in GFP-labeled adult-generated neurons

**DOI:** 10.3389/fnana.2016.00100

**Published:** 2016-10-18

**Authors:** Carles Bosch, Albert Martínez, Nuria Masachs, Cátia M. Teixeira, Isabel Fernaud, Fausto Ulloa, Esther Pérez-Martínez, Carlos Lois, Joan X. Comella, Javier DeFelipe, Angel Merchán-Pérez, Eduardo Soriano

**Affiliations:** ^1^Developmental Neurobiology and Regeneration Unit, Department of Cell Biology, Immunology and Neurosciences and Barcelona Science Park, University of BarcelonaBarcelona, Spain; ^2^Centro de Investigación Biomédica en Red Sobre Enfermedades Neurodegenerativas, Instituto de Salud Carlos IIIMadrid, Spain; ^3^Institut de Recerca de l'Hospital Universitari de la Vall d'Hebron (VHIR)Barcelona, Spain; ^4^Laboratorio Cajal de Circuitos Corticales, Centro de Tecnología Biomédica, Universidad Politécnica de Madrid, Campus de MontegancedoMadrid, Spain; ^5^Instituto Cajal (Consejo Superior de Investigaciones Científicas)Madrid, Spain; ^6^Department of Neurobiology, University of Massachusetts Medical SchoolWorcester, MA, USA; ^7^Departament de Bioquímica i Biologia Molecular, Facultat de Medicina, Institut de Neurociències, Universitat Autònoma de BarcelonaBellaterra, Spain; ^8^Departamento de Arquitectura y Tecnología de Sistemas Informáticos, Escuela Técnica Superior de Ingenieros Informáticos, Universidad Politécnica de MadridMadrid, Spain; ^9^Institució Catalana de Recerca i Estudis Avançats AcademiaBarcelona, Spain

**Keywords:** dendritic spines, synapses, 3D-reconstruction, electron microscopy, FIB/SEM, adult neurogenesis

A sentence in the description of **Figures 5A–D** in the results section of Bosch et al. (2015) contained a minor error, which we hereby rectify (page 7, section “*Three-Dimensional Analysis of Input Synapses onto Mature Adult-Generated Granule Cells*”, paragraph 3, lines 3–5).

This modification does not alter any of the results or claims arisen in the original article, while adds coherence across the manuscript.

It should read:

“Spine and synapse sizes were distributed with a right-skewed curve, whereas sphericities distributed symmetrically around the means (**Figures 5A–D**).”

## Author contributions

Designed the project: CB, JD, AMe, ES; performed experiments: CB, AMa, NM, CT, IF, FU, EP, AMe; contributed with reagents/materials/analyses tools: CB, CT, IF, FU, EP, CL, JC, JD, AMe; analyzed the data: CB, AMa, ES; discussed the results and interpreted the data: CB, AMa, NM, CT, IF, FU, EP, CL, JC, JD, AMe, ES; wrote the article: CB, JDF, AMe, ES.

### Conflict of interest statement

The authors declare that the research was conducted in the absence of any commercial or financial relationships that could be construed as a potential conflict of interest.
