# Supplementary figures and images for: FIB/SEM technology and high-throughput 3D reconstruction of dendritic spines and synapses in GFP-labeled adult-generated neurons
Source: Front Neuroanat. 2015 May 21;9:60. doi: 10.3389/fnana.2015.00060 (PMC4440362; doi:10.3389/fnana.2015.00060)

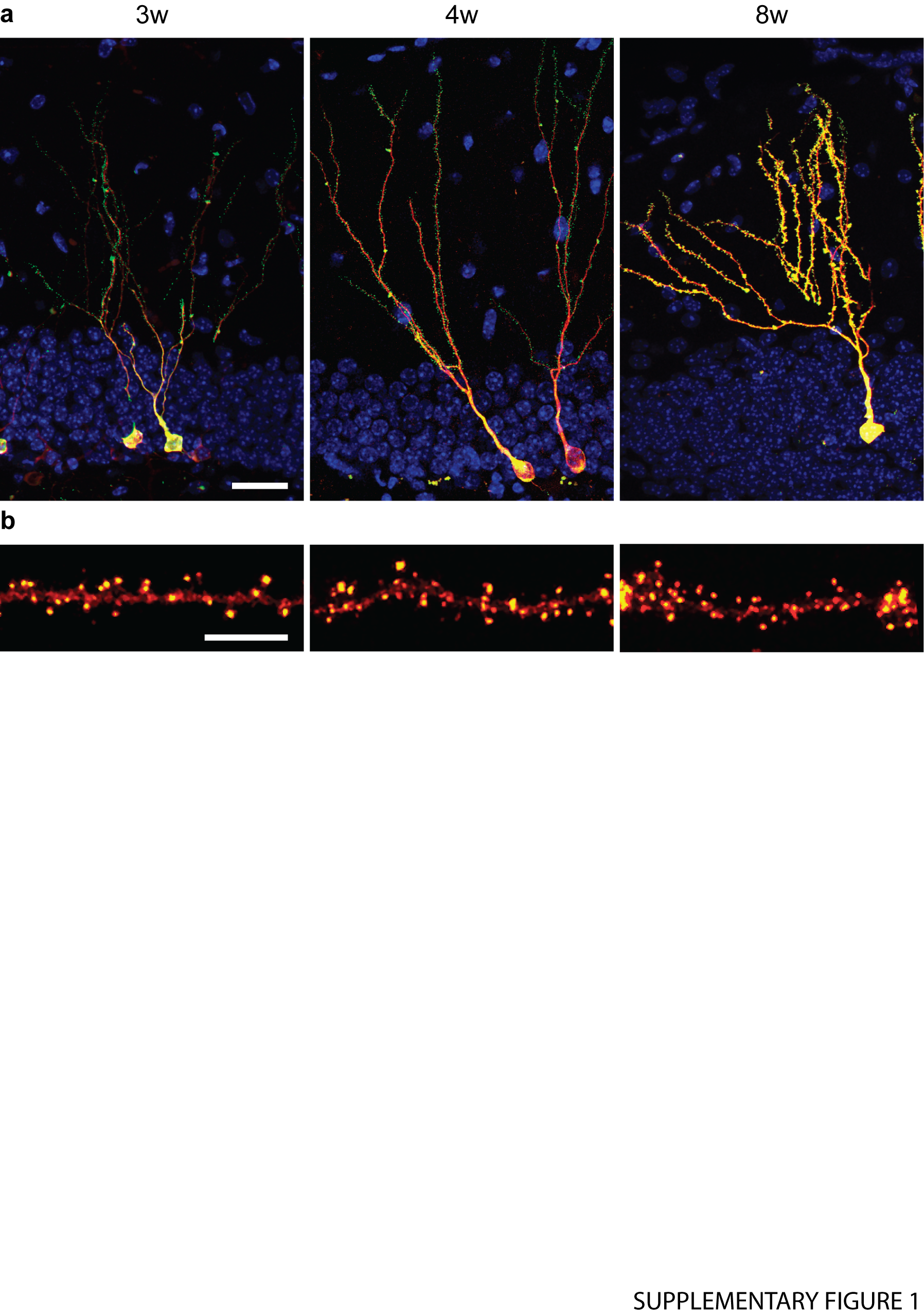

Supplement: Supplementary Figure 1 — Dendritic spines and postsynaptic densities visualized in developing GCs. Newborn GCs were labeled with a retroviral vector expressing PSD95 and visualized at 3, 4 and 8 weeks post-injection. (A) Low magnification views of retrovirally labeled adult-born GCs. (B) Confocal reconstructions of GC dendritic segments showing spines (red) and postsynaptic densities (yellow). Note the increase in spines concomitant to post-injection times. Scale bars are 20 μm in (A) and 5 μm in (B). Abbreviations: GCL, granule cell layer; ML, molecular layer; 3w, 3 weeks post-infection. [file Image1.TIF]

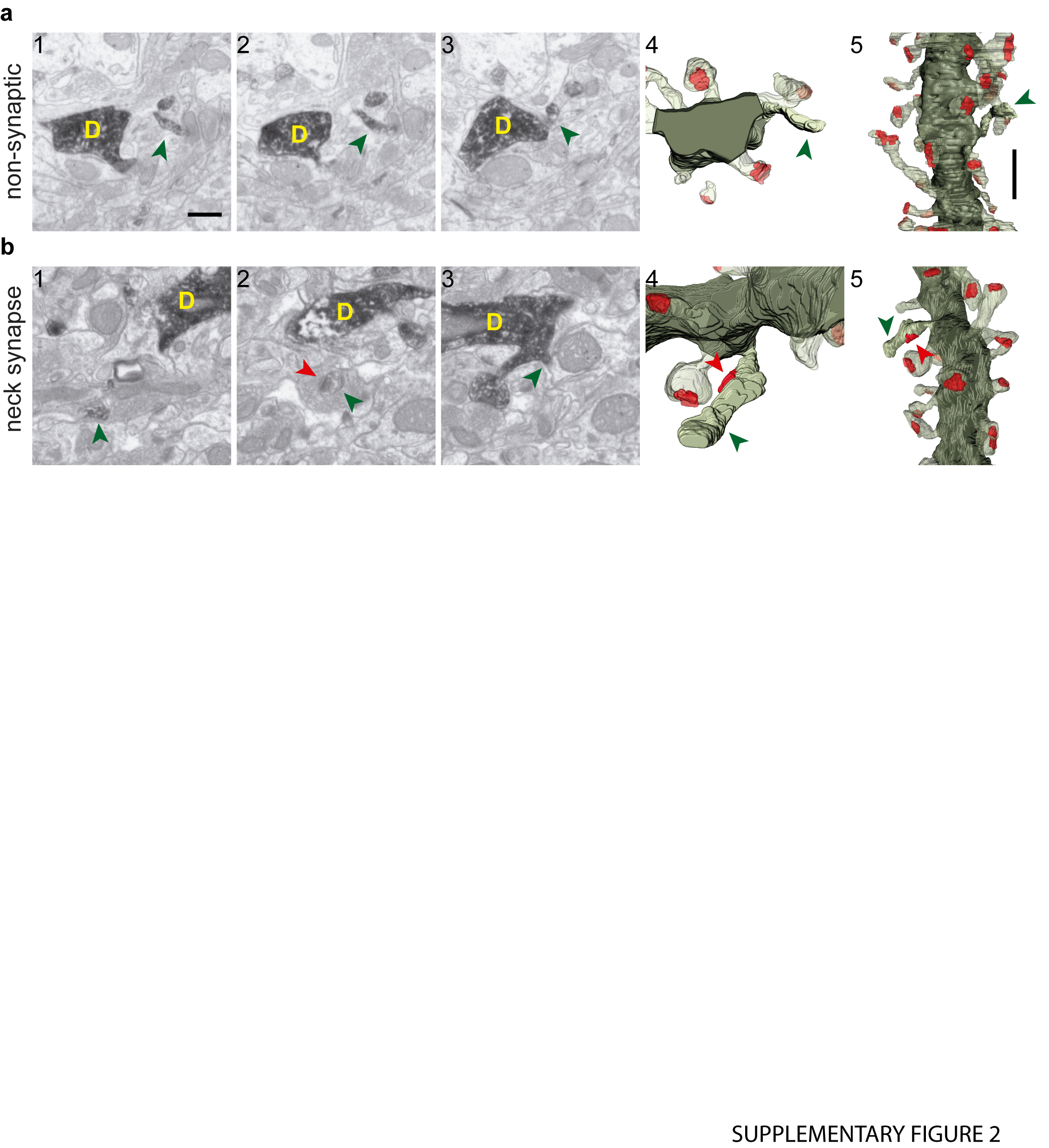

Supplement: Supplementary Figure 2 — Types of dendritic spines in 8-week-old GFP/DAB-labeled GCs as reconstructed with FIB/SEM microscopy. Examples show a non-synaptic spine (A) and a spine receiving synaptic contact in the neck (B). The images on the left show three selected serial planes (1-3) of the spines depicting the head (green arrowheads), neck, and synaptic contact (red arrowheads), while the images on the right show 3D reconstructions (4,5) of the labeled spines in two different orientations. The dendritic shaft (D) is shown in solid dark green, the spine of interest in solid pale green, and its synapse in solid red. Neighboring spines and synapses are shown in light pale green and red, respectively. Scale bar in (A1) is 0.5 μm and applies to (A1-4, B1-4); scale bar in (A5) is 1 μm and applies to (B5). [file Image2.TIF]

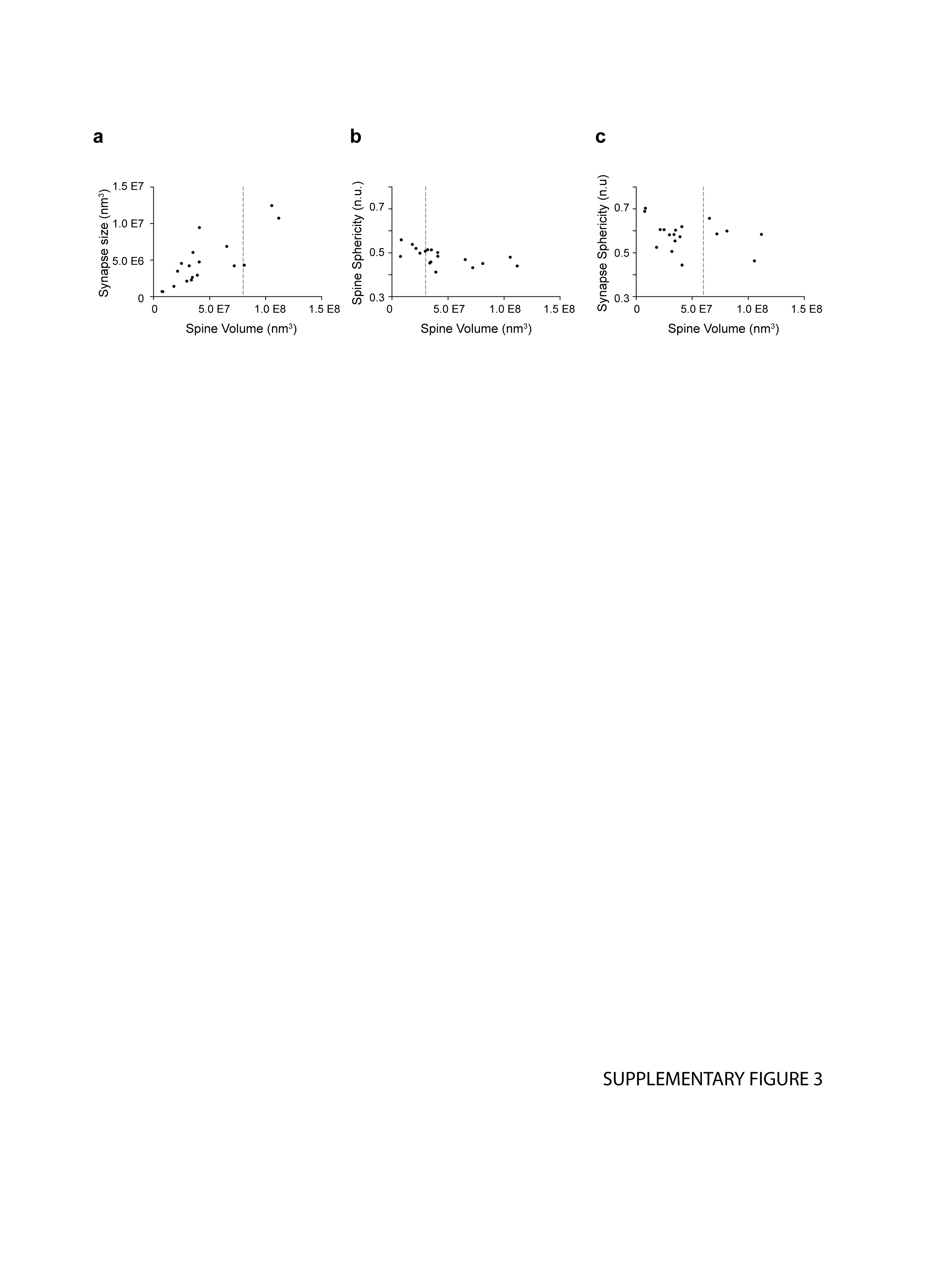

Supplement: Supplementary Figure 3 — Correlation of 3- to 4-week-old GC spine volume with morphometric parameters. (A-c) Plots showing correlation of individual spine volumes with synapse size (Spearman r=0.8060, p<0.001) (A), spine sphericity (Spearman r=− 0.6718, p<0.01) (B), and synapse sphericity (non-significant correlation, Spearman r= −0.29) (c). Spine volume thresholds observed in the 8-9 week group are illustrated by gray dashed lines. [file Image3.TIF]
