# Supplementary material for: FIB/SEM technology and high-throughput 3D reconstruction of dendritic spines and synapses in GFP-labeled adult-generated neurons
Source: Front Neuroanat. 2015 May 21;9:60. doi: 10.3389/fnana.2015.00060 (PMC4440362; doi:10.3389/fnana.2015.00060)
Supplement: Supplementary Table 1 — Numbers of analyzed dendritic spines and presynaptic boutons and their classification. [file Table1.DOC]

**Table 1.** Numbers of analyzed dendritic spines and presynaptic boutons and their classification.

|  | | **3/4 week** | | **8/9 week** | | **Total** | |
| --- | --- | --- | --- | --- | --- | --- | --- |
|  |  | **N** | **%** | **N** | **%** | **N** | **%** |
| **Spines** ^(a)^ |  | 28 |  | 271 |  | 299 |  |
| **3D-reconstructed spines** |  | 22 | 78% | 226 | 83% | 248 | 83% |
| **Non-synaptic spines** |  | 0 | 0% | 5 | 2% | 5 | 2% |
|  | |  |  |  |  |  |  |
| **Spine types** ^(a)^ | Thin | 12 | 48% | 93 | 43% | 105 | 44% |
|  | Mushroom | 6 | 24% | 44 | 20% | 50 | 21% |
|  | Filopodia | 6 | 24% | 36 | 17% | 42 | 18% |
|  | Branched | 1 | 4% | 32 | 15% | 33 | 14% |
|  | Stubby | 0 | 0% | 10 | 5% | 10 | 4% |
|  |  |  |  |  |  |  |  |
| **Synapses** |  | 27 |  | 244 |  | 271 |  |
|  |  |  |  |  |  |  |  |
| **Presynaptic bouton types** | SSBs | 6 | 24% | 63 | 28% | 69 | 28% |
|  | MSBs | 19 | 76% | 162 | 72% | 181 | 72% |

^(a)^ Individual spines sharing a neck were grouped and counted as a branched spine in the spine type analysis.
