# Supplementary material for: FIB/SEM technology and high-throughput 3D reconstruction of dendritic spines and synapses in GFP-labeled adult-generated neurons
Source: Front Neuroanat. 2015 May 21;9:60. doi: 10.3389/fnana.2015.00060 (PMC4440362; doi:10.3389/fnana.2015.00060)
Supplement: Supplementary Table 2 — Statistical analysis of correlations between spine and synapse morphometric parameters in 8-9-week-old GCs. [file Table2.DOC]

**Table 2.** Statistical analysis of correlations between spine and synapse morphometrical parameters at 8-9 week-old GCs.

a) Correlation analysis of spine volume against other variables

|  | **All spines** | | **Spine volume**  **threshold** | **Spines < threshold** | | **Spines > threshold** | |
| --- | --- | --- | --- | --- | --- | --- | --- |
|  | **P**^(a)^ | **Spearman r** |  | **P** | **Spearman r** | **P** | **Spearman r** |
| **Synapse size** | *** | 0.7414 | 8 E7 nm^3^ | *** | 0.7065 | ns | -- |
| **Spine sphericity** | *** | -0.3566 | 3 E7 nm^3^ | *** | -0.3782 | ns | -- |
| **Synapse sphericity** | *** | -0.5016 | 6 E7 nm^3^ | *** | -0.4303 | ns | -- |

^(a)^ *p < 0.05; **p < 0.01; ***p < 0.001; Spearman test. Correlation analysis was performed for spines above and below the spine volume thresholds indicated. Note that for spines with sizes above these thresholds, the correlations were no longer significant.

b) Regression analysis of binned spine volume against other variables (below threshold) for 8-9 week-old GCs

|  | **R^2^** | **P** ^(b)^ | **Slope** | **Y-interceipt** |
| --- | --- | --- | --- | --- |
| **Synapse size** | 0.9529 | *** | 0.07926 | 1.351 E6 |
| **Spine sphericity** | 0.9809 | *** | -3.815 E-9 | 0.6235 |
| **Synapse Sphericity** | 0.8275 | *** | -1.341 E-9 | 0.6526 |

^(b)^ *p < 0.05; **p < 0.01; ***p < 0.001; test whether slopes are significantly non-zero.
